# Supplementary figures and images for: Flow-cytometric microglial sorting coupled with quantitative proteomics identifies moesin as a highly-abundant microglial protein with relevance to Alzheimer’s disease
Source: Mol Neurodegener. 2020 May 7;15:28. doi: 10.1186/s13024-020-00377-5 (PMC7206797; doi:10.1186/s13024-020-00377-5)

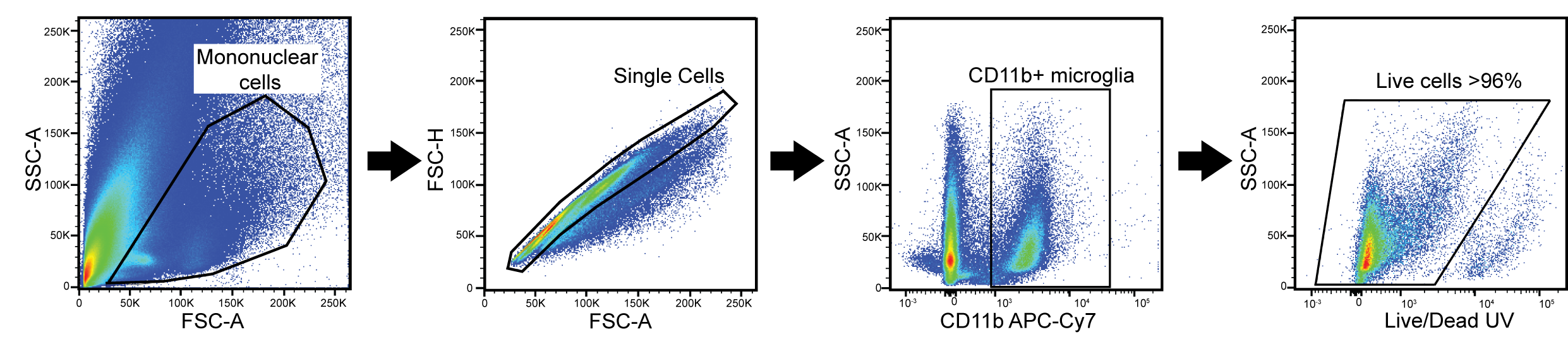

Supplement: Supplementary file 3 — Additional file 3: Figure S1. Viability of mechanically dissociated mouse brain mononuclear cells. Representative flow cytometry data displaying isolation of > 95% live CD11b+ microglia from mechanically dissociated fresh, whole mouse brian (N = 4) following percoll density centrifugation. [file 13024_2020_377_MOESM3_ESM.png]

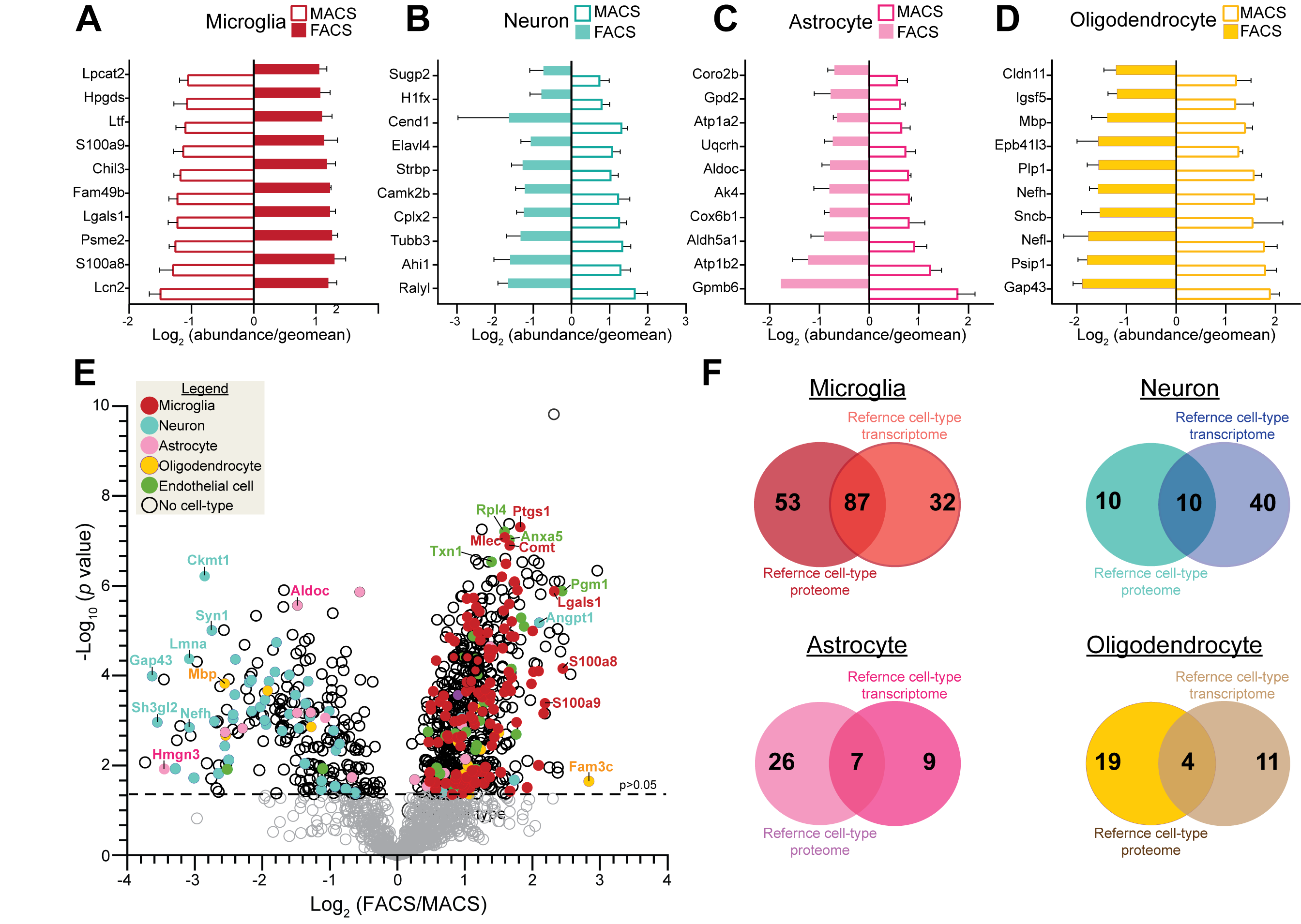

Supplement: Supplementary file 4 — Additional file 4: Figure S2. Enrichment of microglial and endothelial specific proteins by FACS. A-D Histograms displaying top 10 differentially expressed A microglia, B neuron, C astrocyte, and D oligodendrocyte cell-type proteins (defined by Sharma et al. [22]) in FACS-isolated microglia proteome and MACS-enriched microglia proteome. The Y-axis shows list of proteins, X-axis shows Log2-transformed normalized abundance (abundance/row geomean), and error bars represent ± SEM. E Volcano plot displaying the distribution of differentially expressed proteins between FACS-isolated and MACS-enriched microglia proteomes. Cell-type enrichment defined by a reference cell-type transcriptome, Zhang et al. [11], shows significant enrichment of microglial and endothelial specific proteins in the FACS proteome (p < 0.05, Unpaired t-test). Red dots = microglia, turquoise dots = neuron, pink dots = astrocyte, yellow dots = oligodendrocyte, green dots = endothelial cell. Grey dots represent differentially expressed proteins with a p > 0.05. Log2 fold-change is shown on the X-axis, −Log10(p-value) is shown on the Y-axis, and horizontal dotted line indicates p = 0.05. F Venn diagrams comparing number of cell-type specific proteins defined by our two cell-type enrichment analyses: reference cell-type proteome: Sharma et al. [22] and reference cell-type transcriptome: Zhang et al. [11]. [file 13024_2020_377_MOESM4_ESM.png]

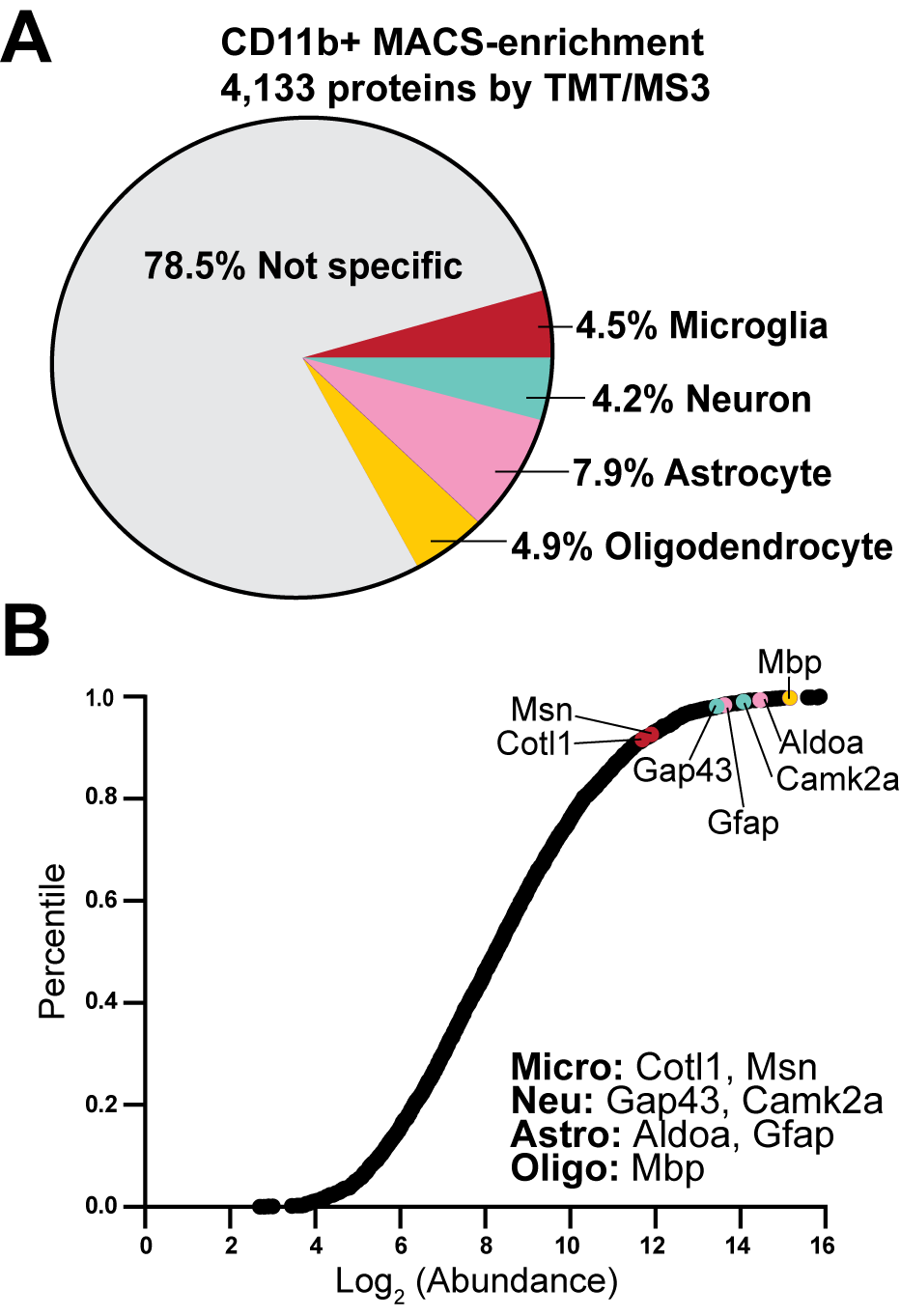

Supplement: Supplementary file 5 — Additional file 5: Figure S3. Contamination of non-microglial proteins in MACS-enriched microglia proteome. A Pie chart displaying distribution of cell-type enrichment in previously published microglial proteome, Rangaraju et al. [25], where 4.5% of the 4133 quantified proteins are microglial-specific while nearly 17% of the proteins are from other brain cell-types. B Highly-abundant proteins in a MACS microglial proteomic study [25] included non-microglial proteins such as Mbp, Aldoa, Gfap, and Camk2a. Micro = microglia, Neu = neuron, Astro = astrocyte, Oligo = oligodendrocyte. [file 13024_2020_377_MOESM5_ESM.png]

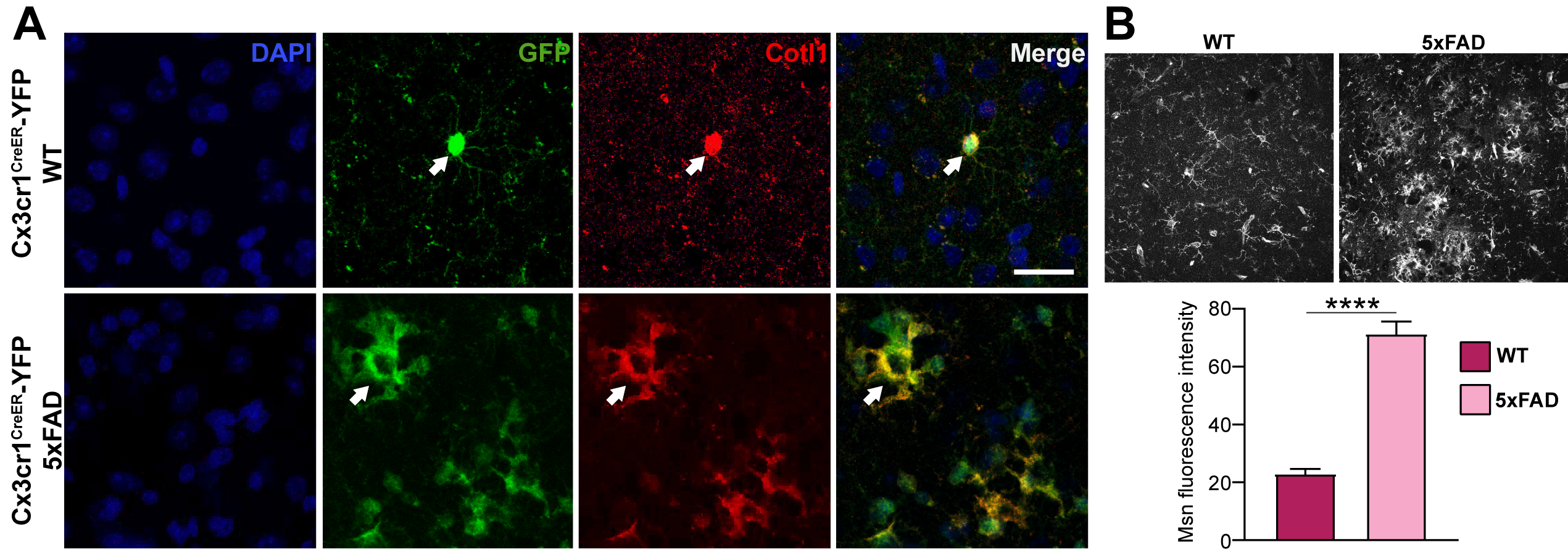

Supplement: Supplementary file 6 — Additional file 6: Figure S4. Validation of Cotl1 in microglia. A Representative immunofluorescence images of Cx3cr1CreER-YFP-WT (N = 4) and Cx3cr1CreER-YFP-5xFAD (N = 6) mouse cortex stained for GFP (microglia) and Cotl1. Arrow indicates microglia immunopositive for GFP (to detect microglia) and Cotl1. B Representative images of 9–10 month old WT and 5xFAD cortex used for quantitative analysis of Msn fluorescence intensity shown in a histogram below. Unpaired t-test, ****p < 0.0001. [file 13024_2020_377_MOESM6_ESM.png]

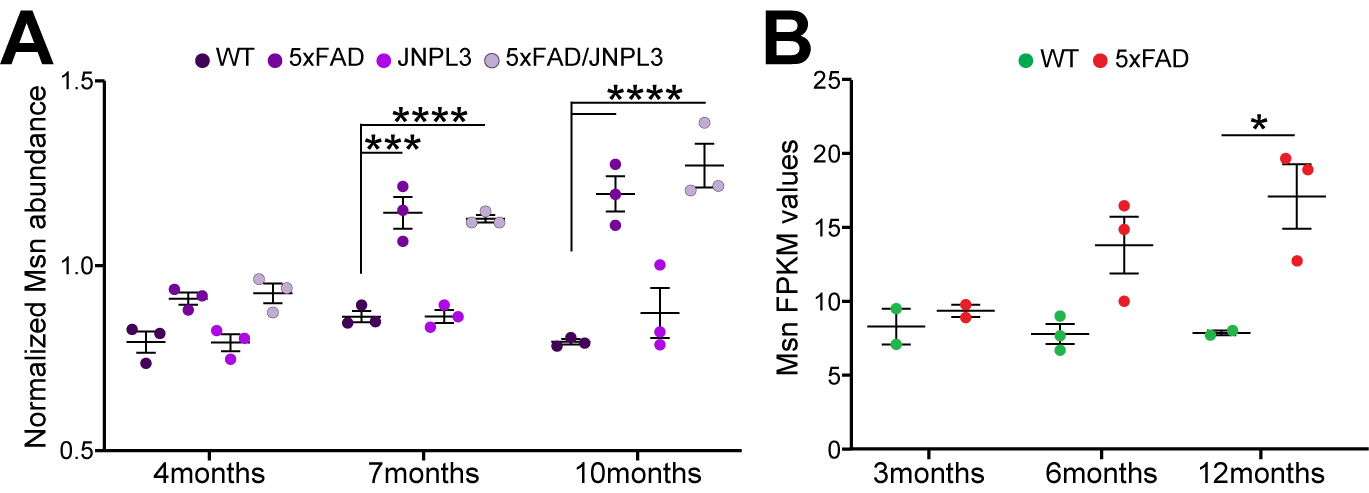

Supplement: Supplementary file 7 — Additional file 7: Figure S5. Moesin protein levels are increased in human AD and FTLD-TDP brain. A Msn protein abundance in dorsolateral prefrontal cortex post-mortem brain tissue of control (N = 43), AD (N = 47), FTLD-TDP (N = 29), ALS (N = 54), and PD/PDD (N = 76) cases; UPENN cohort. Protein abundance was measured with LFQ [40]. One-way ANOVA, Tukey post hoc: *p < 0.05,***p < 0.001. B Msn protein abundance in precuneus post-mortem brain tissue of control (N = 13), AsymAD (N = 13), and AD (N = 20) cases; BLSA cohort. Protein abundance was measured with LFQ [40]. One-way ANOVA, Tukey post hoc: *p < 0.05,***p < 0.001. C Msn protein abundance in laser capture microdissected Aβ plaques from human post- mortem brain tissue of rapid progression AD (N = 22) and sporadic AD (N = 22) cases. Protein abundance was measured with LFQ by Drummond et al. [42]. Unpaired t-test: ***p < 0.001. [file 13024_2020_377_MOESM7_ESM.png]

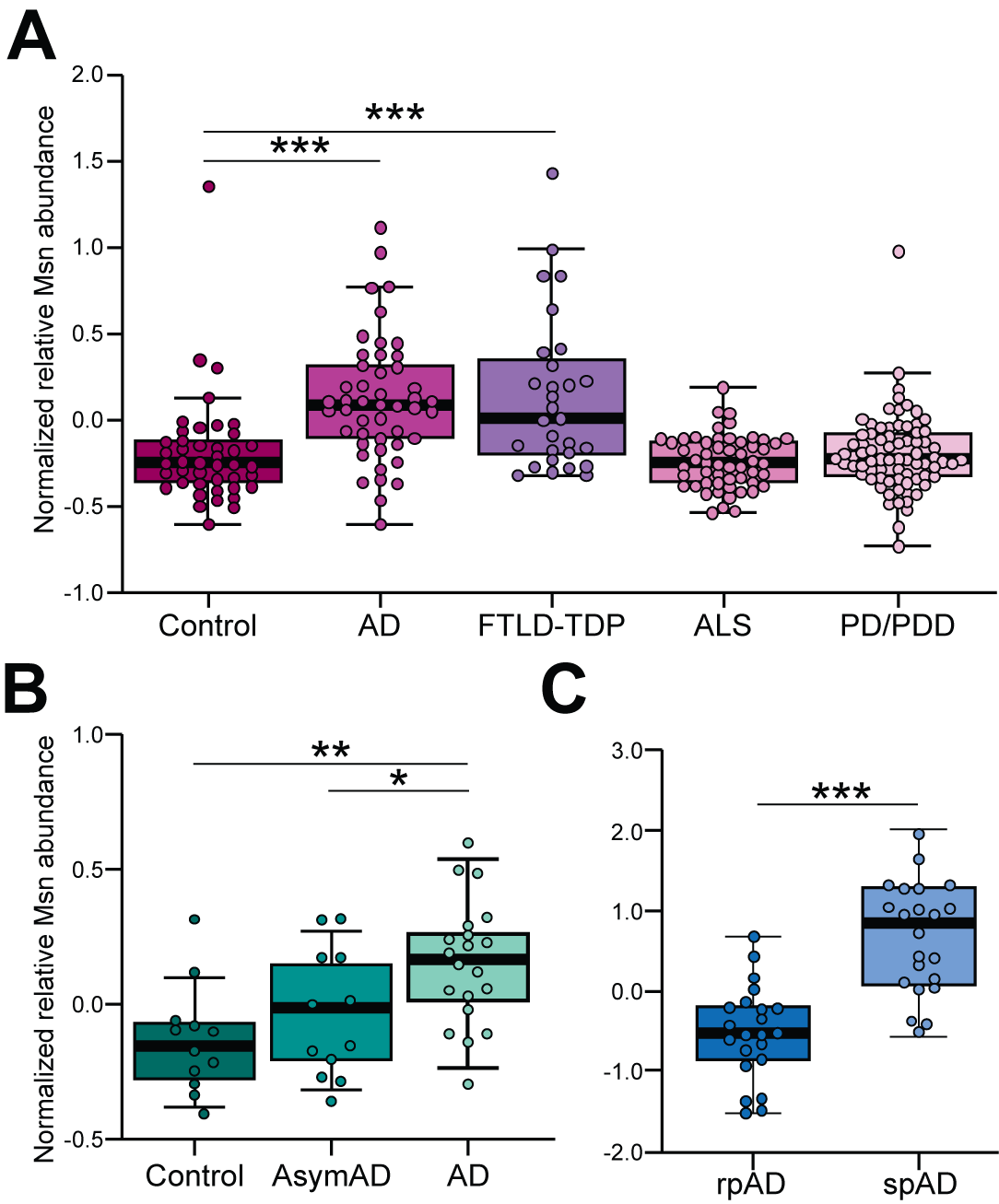

Supplement: Supplementary file 8 — Additional file 8: Figure S6. Moesin protein and mRNA levels increase with age in 5xFAD brain. A Normalized relative Msn protein abundance in brains of wild-type (WT), 5xFAD (Aβ pathology), JNPL3 (Tau pathology), and a cross of 5xFAD and JNPL3 (Aβ & tau pathologies) mice at 4 months, 7 months, and 10 months of age (N = 3/age/genotype). Protein abundance obtained by TMT-MS [43]. B Relative Msn expression, shown as FPKM values, in brains of WT and 5xFAD mice at 3 months, 6 months, and 12 months of age (N = 2–3/age/genotype) from a previously published study [44]. Error bars represent ± SEM. One-way ANOVA, Tukey post hoc analysis: *p < 0.05, ***p < 0.001, ****p < 0.0001. [file 13024_2020_377_MOESM8_ESM.png]
